# Feasibility and safety of early discharge after robot-assisted uro-oncologic surgery using wearable telemonitoring

**DOI:** 10.1007/s11701-026-03696-9

**Published:** 2026-08-31

**Authors:** Andrea Fuschi, Manfredi Bruno Sequi, Antonio Nacchia, Yazan Al Salhi, Fabio Maria Valenzi, Paolo Pietro Suraci, Alessandro Corsini, Damiano Graziani, Riccardo Lombardo, Flavia Carbone, Giuseppe Candita, Filippo Gianfrancesco, Paolo Benanti, Giovanni Di Gregorio, Luca Erra, Sebastiano Sciarretta, Cosimo De Nunzio, Arnaldo Usai, Antonio Luigi Pastore, Antonio Carbone

**Affiliations:** 1 Urology Unit, Department of Medico-Surgical Sciences and Biotechnologies, Faculty of Pharmacy and Medicine, Sapienza University of Rome, Via Franco Faggiana 1668, 04100 Latina, Italy; 2 I.C.O.T. Hospital, Urology Unit, Latina, Italy; 3 Department of Urology, Sant’Andrea Hospital, Sapienza University of Rome, Via di Grottarossa 1035/1039, 00189 Rome, Italy; 4 Translational Innovation in Medicine and Surgery, Department of Medical- Surgical Sciences and Translational Medicine, Sapienza University of Rome, Rome, Italy; 5 Department of Mechanical and Aerospace Engineering, Sapienza University of Rome, Rome, Italy; 6 Department of Medical-Surgical Sciences and Biotechnologies, Sapienza University of Rome, Latina, Italy; 7 IRCCS NeuroMed, Pozzilli, Italy; 8 Digital Uniforms - Solutions and Technologies, Via Mazzini n.87, SS 07041 Alghero, Italy

## Abstract

Early discharge after robot-assisted uro-oncologic surgery remains limited by concerns regarding patient safety and the timely detection of postoperative complications. Wearable telemonitoring may facilitate earlier discharge while maintaining clinical surveillance during recovery. In this multicenter prospective study, 86 patients undergoing robot-assisted radical prostatectomy, partial nephrectomy, or radical nephrectomy were allocated to a control group (C, *n* = 43) receiving standard postoperative care or a wearable group (W, *n* = 43) managed through an early-discharge telemonitoring protocol. Patients in group W were discharged 48 h earlier and monitored remotely using a wearable sensor-equipped shirt and smartphone application. The system continuously recorded respiratory rate, heart rate, body temperature, and single-lead electrocardiographic data, while blood pressure, oxygen saturation, and blood glucose values were entered manually. The primary endpoint was the feasibility and safety of early discharge. Secondary endpoints included length of stay (LoS), early unscheduled hospital access, postoperative complications, readmissions, and usability assessed using the Mobile Health App Usability Questionnaire (MAUQ). The telemonitoring protocol enabled a planned 48-hour earlier discharge while maintaining comparable postoperative outcomes (2.8 ± 0.4 vs. 4.7 ± 0.8 days, *p* < 0.001). Early unscheduled hospital access was significantly lower in group W than in group C (2.3% vs. 25.6%, *p* < 0.01). Minor postoperative complications (Clavien-Dindo grade < III) occurred in 7.0% and 11.6% of patients in groups W and C, respectively (*p* = 0.71), while no major complications (Clavien-Dindo grade ≥ III) were observed. The monitoring system generated 68 alerts, leading to teleconsultation and, when clinically indicated, in-person evaluation. Three patients were readmitted following critical alerts related to arrhythmias or hypertensive crises. Adherence to monitoring was high, and technical issues (16.3%) were resolved without data loss. The mean MAUQ score was 95.3 ± 11.4, indicating high user satisfaction. Early discharge supported by wearable telemonitoring appears feasible and safe in carefully selected patients undergoing robot-assisted uro-oncologic surgery. The approach was associated with fewer unscheduled postoperative hospital visits, high patient satisfaction, and effective remote surveillance during recovery. Larger randomized studies are warranted to confirm these findings and evaluate the long-term clinical and economic impact of telemonitoring-based recovery pathways.

Keywords: Remote monitoring; Telemonitoring; Wearable devices; Telemedicine; Early discharge; Robot-assisted surgery; Urologic oncology; Postoperative care.

**Supplementary Information:**

The online version contains supplementary material available at https://doi.org/10.1007/s11701-026-03696-9.

## Introduction

 The concept of telemedicine dates back to 1877, when physicians in adjacent areas created the first telephone exchange system to facilitate communication with local pharmacies [1]. Numerous efforts have been made to advance information-based technologies for healthcare delivery, but widespread clinical implementation has remained limited due to clinical, financial, and technical barriers [1]. Despite these challenges, telemedicine has become integral to many medical specialties, including psychiatry [2, 3], ophthalmology [4], dermatology [5], neurology [6], and cardiology [7]. The global COVID-19 pandemic in 2020 catalyzed the rapid adoption of telemedicine. Hospitals overwhelmed with COVID-19 cases diverted resources from routine care, significantly reducing their capacity for oncology and other non-pandemic-related patients [8]. This crisis accelerated the development and implementation of remote patient monitoring systems, including wearable devices, to manage infected patients effectively [9]. During this period, initiatives such as the European Union’s €750 billion Next Generation EU (NGEU) program were launched to support public health and mitigate the pandemic’s socioeconomic impact, with substantial funding allocated to healthcare digitization and telemedicine. Italy alone is set to receive up to €20 billion for these advancements [10]. Thanks to these economic and technical efforts, today’s market offers a wide range of telemedicine applications. The primary applications include televisits, Teleassistance, telemonitoring, and teleconsultation [11]. Their success largely depends on healthcare providers’ ability to integrate these services into operational contexts with varying levels of technological sophistication and personnel training in digital healthcare systems [12]. In recent years, wearable devices have garnered increasing attention for their potential to revolutionize healthcare [13]. Our study investigates the feasibility of early discharge for patients undergoing major robot-assisted uro-oncologic surgery, utilizing wearable devices and web-based software for telemonitoring.

## Materials and methods

### Study design and inclusion criteria

In this multicenter prospective study, patients undergoing robot-assisted radical prostatectomy (RARP), radical nephrectomy (RARN), and partial nephrectomy (RAPN), for urologic malignancies were treated at the Urology Unit of ICOT Hospital in Latina and the Azienda Ospedaliera Universitaria Sant’Andrea. Admission to the study required meeting the predefined criteria (internet access, smartphone availability, and the presence of a caregiver). Participation was voluntary, and patients were enrolled only after providing written informed consent. Patients who agreed to participate received a wearable telemonitoring system, a patented technology invented by Dr. G. Arnaldo Usai in the e-textile domain. These patients were discharged 48 h earlier than the standard postoperative schedule and were remotely monitored throughout the early postoperative recovery period. The inclusion criteria were patients with urological malignancies who were eligible for robot-assisted surgery, had access to the internet and a smartphone, and had a caregiver living with or close to the patient during the study. Patients who failed pre-operative assessments were excluded from the study.

### Protocol

Patients were consecutively enrolled from August 2023 to October 2024 and were divided into two groups: a control group (C) and a study group (W) based on their willingness and written consent to use the wearable monitoring system. Because participation in the telemonitoring pathway required patient acceptance and caregiver involvement, allocation was based on patient preference rather than randomization. This study was approved by the Ethical Committee of the Azienda Ospedaliera Sant’Andrea (Number # prot.n.34SA/2023, rif. CE7081/2023, January 9th, 2023) and adhered to the Declaration of Helsinki. The telemonitoring protocol, along with the device’s features and functionality, was thoroughly explained to the patients and their caregivers during the preoperative urological consultation, and written consent was obtained. All participants cleared for surgery were admitted 1 day prior to surgery. Patients in the W group were fitted with a sensor-equipped shirt to ensure proper sizing, pairing, and device functionality, and to allow the technical team to verify signal quality and connectivity. This preoperative fitting also familiarized patients and caregivers with the system and enabled the resolution of any technical issues before discharge. Preoperative baseline parameters were collected, including sex, age, body mass index (BMI), smoking status, diabetes mellitus (DM), hypertension (HT), use of anticoagulant and/or antiplatelet drugs, presence of cardiovascular comorbidities, Charlson Comorbidity Index (CCI), heart rate (HR), mean arterial pressure (MAP), oxygen saturation (SpO_2_), Self Monitoring of Blood Glucose (SMBG), and hemoglobin (Hb). All surgical procedures were performed under general anesthesia using the “Da Vinci X” system (Intuitive Surgical, Sunnyvale, CA, USA) by expert robotic surgeons (each with > 200 oncologic robotic cases). All procedures were performed using a standardized four-arm robotic configuration with monopolar curved scissors in the right robotic arm, Maryland bipolar forceps in the left robotic arm, and a ProGrasp™ forceps in the fourth robotic arm for tissue traction and countertraction. In RARP, pedicle dissection and nerve-sparing, when indicated, were performed using a conventional robotic bimanual traction-countertraction technique. In RARN, the renal hilum was routinely identified and dissected, and vascular control was achieved with Hem-o-lok^®^ clips. In RAPN, the renal hilum was systematically identified and dissected before tumor excision, with a preferential off-clamp approach, and selective arterial clamping reserved for tumors > 5 cm or when deemed necessary based on tumor complexity and intraoperative findings. Following tumor excision, renorrhaphy was performed using a standardized two-layer closure with running 3 − 0 Monocryl for the inner layer and 2 − 0 Monocryl for the outer cortical layer. Perioperative and postoperative parameters included operative time (OT), estimated blood loss (EBL), HR, MAP, SpO_2_, SMBG, body temperature (T), Hb, length of hospital stay (LoS), and hospital readmissions. The primary endpoint was feasibility and safety of early discharge supported by wearable telemonitoring. Feasibility was assessed based on protocol adherence, successful completion of the monitoring period, the system’s technical reliability, and the proportion of patients who completed postoperative recovery at home without protocol-related discontinuation. Safety was assessed through postoperative complications, hospital readmissions, and early unscheduled hospital access. Discharge criteria were identical for both groups: hemodynamic stability, independent ambulation, tolerance of oral intake, adequate pain control, and absence of major complications. Patients in Group W were discharged 48 h earlier than the institution’s standard postoperative pathway, provided that all predefined discharge criteria were met. Early unscheduled hospital access was defined as any unplanned in-person evaluation in the emergency department, outpatient clinic, or hospital ward that occurred before the scheduled postoperative follow-up visit. Telephone consultations alone were not considered hospital access events.

### Telemonitoring

**Fig. 1:**
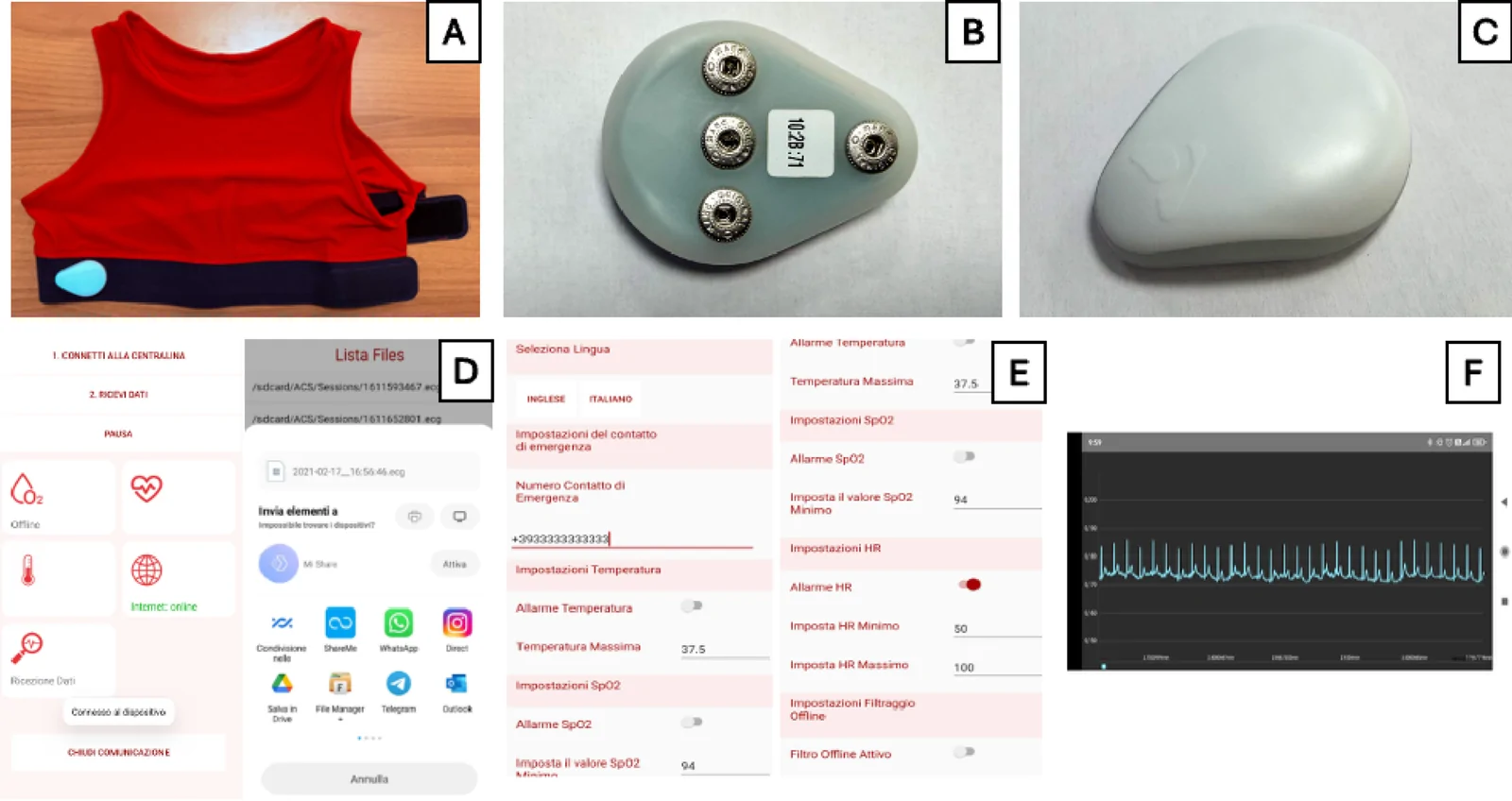
Wearable telemonitoring platform employed for early discharge after robot-assisted uro-oncologic surgery. (**A**) Smart garment with integrated textile electrodes and a sensor band. (**B**) Internal aspect of the monitoring unit showing electrode contacts. (**C**) External aspect of the wireless monitoring unit. (**D**) Smartphone application interface for secure transmission and sharing of physiological data. (**E**) Alert management screen for customizing physiological thresholds and emergency settings. (**F**) Real-time visualization of a single-lead ECG signal acquired by the wearable device and transmitted to the remote monitoring platform

Telemonitoring was carried out on patients of group W with the help of a sensor-equipped shirt and a mobile app (Fig. 1) responsible for the management of summary information on the patient side following e-health principles and strictly coherent to GDPR prescriptions, with respect to the progress of the patient journey, and for the reporting of possible anomalies (outside thresholds). All patients and their respective caregivers were informed of the need to wear the shirt 10 h a day for 8 to 20 days. The monitoring duration was selected to provide continuous physiological tracking during daily activity while ensuring patient comfort and adherence. The total monitoring period of 8–20 days covered the early postoperative phase, when most physiologic variations and potential complications typically occur [14–16]. The shirt was provided to the patients on the day of discharge and paired with the app via Bluetooth. The shirt enabled continuous monitoring of RR, HR, T°, and heart rhythm [through a single-lead electrocardiogram (ECG) embedded in the shirt], while patients manually entered the remaining vital signs [blood pressure (BP), SpO_2_, and SMBG] twice a day in the app. The monitoring data were automatically transmitted via a secure cloud-based system to a web platform accessible by the study investigators. A color-coded system was used to classify parameters: Blue (normal range), Yellow (moderate anomalies), and Red (critical anomalies requiring immediate intervention). For HR, values of 100–120 beats per minute (bpm) or 50–59 bpm triggered Yellow alerts, while values > 120 bpm or < 50 bpm triggered Red alerts. RR of 20–24 breaths per minute (breaths/pm) were flagged as Yellow, and ≥ 25 or < 10 breaths/pm as Red. A body T between 37.5 and 38.0 °C was classified as Yellow; values > 38.0 °C or < 35.5 °C as Red; BP alerts were based on systolic and diastolic thresholds: Yellow for systolic 140–159 or 90–99 mmHg, and diastolic 50–59 or 90–99 mmHg; Red for systolic ≥ 160 or < 90 mmHg, and diastolic ≥ 100 or < 50 mmHg. SpO₂ between 92 and 94% was considered Yellow, and < 92% Red. SMBG levels of 140–180 mg/dL or 60–69 mg/dL triggered Yellow alerts, while > 180 or < 60 mg/dL triggered Red alerts. The system was designed to identify physiological deviations rather than to diagnose specific surgical complications. Each alert was reviewed by the medical team to determine whether the underlying cause could be surgical or non-surgical. At the end of the monitoring period, patients completed the Italian-translated version of the Mobile Health App Usability Questionnaire (MAUQ) [17]. The MAUQ employs a 7-point scale for each of its 21 items, with total scores ranging from 21 to 147. Responses with scores ≥ 4 were considered positive, and the app was deemed satisfactory if the total score was ≥ 84. The study workflow, including patient enrollment, allocation, monitoring completion, alerts, and readmissions, is summarized in Supplementary Figure S1.

### Statistical analysis

Statistical analyses were conducted using IBM SPSS Statistics, version 29.0 (IBM Corp., Armonk, NY, USA). Normality of data distribution was verified using the Shapiro-Wilk test. Continuous variables were expressed as mean ± standard deviation (SD) or median (interquartile range, IQR), while categorical variables were expressed as frequencies and percentages. Comparisons between groups were made using the Student’s t-test or Mann-Whitney U-test for continuous data, and the chi-square or Fisher’s exact test for categorical data, as appropriate. A two-tailed p-value < 0.05 was considered statistically significant.

## Results

### Baseline characteristics, Perioperative, and Postoperative outcomes

The demographic and preoperative clinical parameters of the two groups (C and W, each with 43 patients) were comparable, as shown in Table 1. Key characteristics, including mean age, BMI, DM, HT, and prevalence of cardiovascular disease, showed no statistically significant differences (all *p* > 0.05). Baseline HR, RR, Blood Glucose, SpO_2_, MAP, and Hb were comparable between the groups, as were the CCI scores, with no statistically significant differences found. Smoking habits were also homogeneous across the cohorts.

**Table 1:** Baseline characteristics, perioperative outcomes, and discharge parameters of patients receiving standard postoperative care (Group C) versus wearable telemonitoring with early discharge (Group W). Data are presented as mean (SD) or percentage. BMI: body mass index; DM: diabetes mellitus; HT: hypertension; CCI: Charlson Comorbidity Index; HR: heart rate; RR: respiratory rate; SMBG: self-monitored blood glucose; SpO₂: peripheral oxygen saturation; MAP: mean arterial pressure; Hb: hemoglobin; EBL: estimated blood loss; RARP: robot-assisted radical prostatectomy; RARN: robot-assisted radical nephrectomy; RAPN: robot-assisted partial nephrectomy

Preoperative variables	Group C	Group W	*p*
Number of Patients	43	43	
Age, Years (SD)	62.3 (8.6)	61.5 (9.4)	0.68
Female, %	11.63	13.95	0.96
BMI, Kg/m2 (SD)	24.8 (2.6)	24.3 (3.2)	0.38
DM, %	18.6	16.28	0.95
HT, %	55.8	48.8	0.66
Smoking Habit, %	30.23	34.88	0.81
Antiplatelet/anticoagulant, %	46.51	48.84	0.96
Cardiovascular disease, %	32.56	37.21	0.83
CCI			0.84
2, %	6.98	4.65	
3, %	11.62	9.3	
4, %	27.9	30.23	
5, %	46.52	53.48	
6, %	6.98	2.3	
Preoperative Vitals			
HR, bpm (SD)	66.9 (6.4)	68.7 (5)	0.15
RR, breaths/pm (SD)	17 (1.4)	16.9 (1.37)	0.89
SMBG, mg/dL (SD)	98.4 (9.2)	99.8 (9.3)	0.92
SpO_2_ (SD)	98.3 (0.55)	98.3 (0.6)	0.75
MAP (SD)	96.8 (3.3)	96.7 (2.9)	0.96
T, °C (SD)	36.7 (0.4)	36.8 (0.3)	0.98
Preoperative Hb, g/dL (SD)	13.81 (1.07)	14.07 (0.94)	0.24

Postoperative variables	Group C	Group W	*p*
Number of Patients	43	43	
Surgical Procedure			0.8
RARP, %	65.12	62.8	
RARN, %	9.3	13.95	
RAPN, %	25.58	23.25	
Operative time	158.2 (22.3)	153 (20.9)	0.27
EBL, mL (SD)	270.9 (40)	258.4 (48)	0.19
Postoperative Hb, g/dL (SD)	12.19 (0.71)	12.48 (0.99)	0.12
Hospital stay, Days (SD)	4.7 (0.8)	2.8 (0.4)	< 0.001
Clavien-Dindo < III, %	11.6	7	0.71
Clavien-Dindo > III, %	0	0	N/A
Vital Signs at Discharge			
HR, bpm (SD)	72.8 (4.2)	72.6 (5.8)	0.95
RR, breaths/pm (SD)	18.1 (1.3)	18.3 (1.37)	0.88
SMBG, mg/dL (SD)	98.8 (8.4)	99.2 (6.8)	0.8
SpO2, % (SD)	98.1(0.8)	98.4 (0.9)	0.06
MAP, mmHg (SD)	98.1 (3.1)	99.1 (3)	0.13
Unscheduled Hospital Access%	25.58	2.32	< 0.01

The procedures performed were similar across groups, with RARP being the most common, followed by RAPN and RARN (overall *p* = 0.80). Operative time was comparable between Group C (158.2 ± 22.3 min) and Group W (153.0 ± 20.9 min; *p* = 0.27). EBL was also similar, averaging 270.9 ± 40.0 mL in Group C and 258.4 ± 48.0 mL in Group W (*p* = 0.19). Postoperative Hb did not differ significantly (12.19 ± 0.71 vs. 12.48 ± 0.99 g/dL, *p* = 0.12). Minor postoperative complications (Clavien-Dindo grade < III) occurred in 11.6% of patients in Group C and 7.0% in Group W (*p* = 0.71). No major complications (Clavien-Dindo grade ≥ III) were observed in either group. The LoS was significantly shorter in Group W (2.8 ± 0.4 days) than in Group C (4.7 ± 0.8 days, *p* < 0.001). Vital signs at discharge were comparable between groups (all *p* > 0.05). Early unscheduled hospital access was significantly lower in Group W.

### Telemonitoring Results

**Fig. 2:**
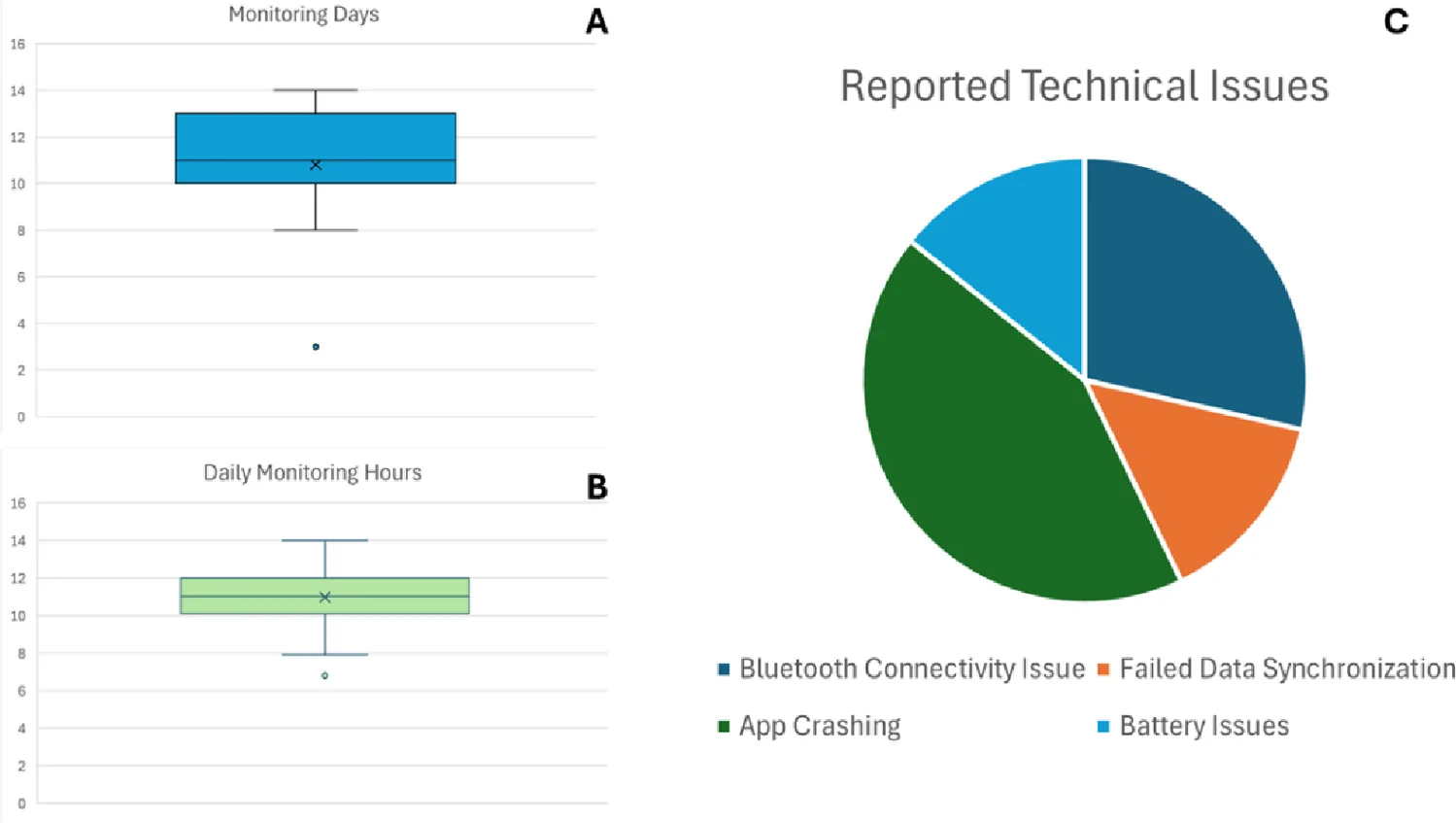
Adherence and system performance among patients monitored after discharge (*n* = 43 in the wearable group). (**A**) Boxplot of monitoring duration (days); median = 11 days, IQR = 10–13 days. (**B**) Boxplot of daily shirt usage (hours); median = 11 h, IQR = 10.1–12 h. (C) Pie chart showing the distribution of technical issues reported (16.3% of participants): Bluetooth disconnection (37.5%), synchronization failure (31.3%), app crash (18.8%), battery limitation (12.5%). All issues were resolved remotely within hours, with no data loss or missed alerts

Patients were monitored for an average of 10.67 ± 2.34 days, with a median duration of 11.0 days [interquartile range (IQR): 10.0–13.0]. The average daily usage of the shirt was 10.98 ± 1.59 h, with a median daily usage of 11.0 h (IQR: 10.1–12.0 h). The recommended wearing time of 10 h per day was achieved by 90.7% of the study population. The remaining 9.3% of patients reported difficulties maintaining the required usage time due to discomfort, technical issues, or personal circumstances. Technical issues, such as Bluetooth connectivity problems, data synchronization issues, battery limitations, and app crashes, were reported by 16.27% of the cohort. Technical support resolved most problems remotely within a short time frame, ensuring no data loss or missed alerts occurred. One patient dropped out of the study due to the onset of atrial fibrillation (AF) that required readmission on the third monitoring day. Figure 2 summarizes the findings related to protocol adherence and technical issues.

**Fig. 3:**
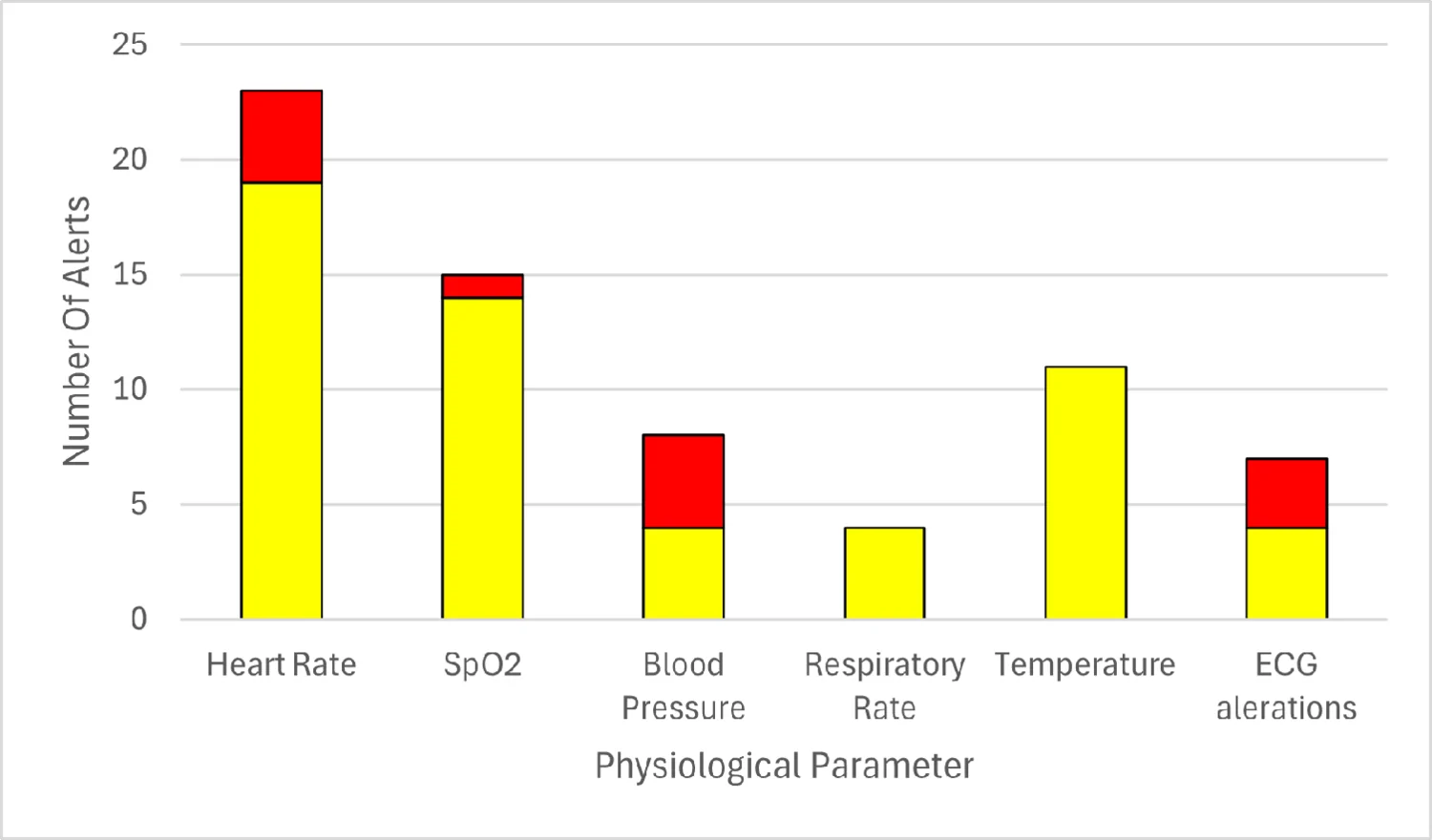
Distribution of alerts recorded by the wearable system. The system signaled physiological deviations but did not directly diagnose surgical complications; each alert was reviewed by the clinical team

The system generated 68 alerts over the study period. These included 56 Yellow and 12 Red alerts. The most detected abnormal parameters included HR (33.82%), SpO₂ (22.05%), T (16.17%), BP (11.76%), RR (5.9%), and ECG alterations (10.3%). Among the ECG alterations, episodes suggestive of bradyarrhythmia and tachyarrhythmia, as well as two cases of AF, were detected. The alerts are shown in Fig. 3.

After immediate teleconsultation with a urologist or cardiologist, 7 cases required in-person evaluation. Among these, 3 patients were readmitted following a critical alert, including 2 for ECG-related abnormalities and 1 for a hypertensive episode. Yellow alerts were generally managed through remote counseling or increased monitoring frequency without requiring hospital evaluation or readmission. Of the three readmitted patients, one developed atrial fibrillation on the third day of monitoring and consequently discontinued the telemonitoring protocol. The remaining two patients had already completed at least 8 days of monitoring before clinically relevant events prompted hospital evaluation and subsequent readmission. Overall, the telemonitoring protocol proved feasible. A total of 90.7% of patients achieved the recommended daily device utilization, 42 of 43 patients successfully completed the planned monitoring period, and all technical issues were resolved without data loss. Only one patient discontinued monitoring prematurely due to an AF-related hospital readmission. Alert breakdown is displayed in Table 2.

**Table 2:** Red Alerts Analysis. A total of 12 patients with Red alerts required an initial teleconsultation, of whom 7 underwent in-person evaluation, and 3 were ultimately readmitted to the hospital. The table summarizes the causes of escalation at each level, with arrhythmias and blood pressure abnormalities accounting for the majority of interventions

Red Alert Outcome	Number of Patients	Main Causes
Teleconsultation	12	Tachycardia (2) Bradycardia (2) SpO₂ Drop (1) ECG alterations (3) BP alterations (4)
In-person evaluation	7	Bradycardia (1) Hypertension (2) Hypotension (1) Tachyarrhythmia (1) Atrial Fibrillation (2)
Hospital readmission	3	Atrial Fibrillation (1) Tachyarrhythmia (1) Hypertension (1)

### Usability

**Fig. 4:**
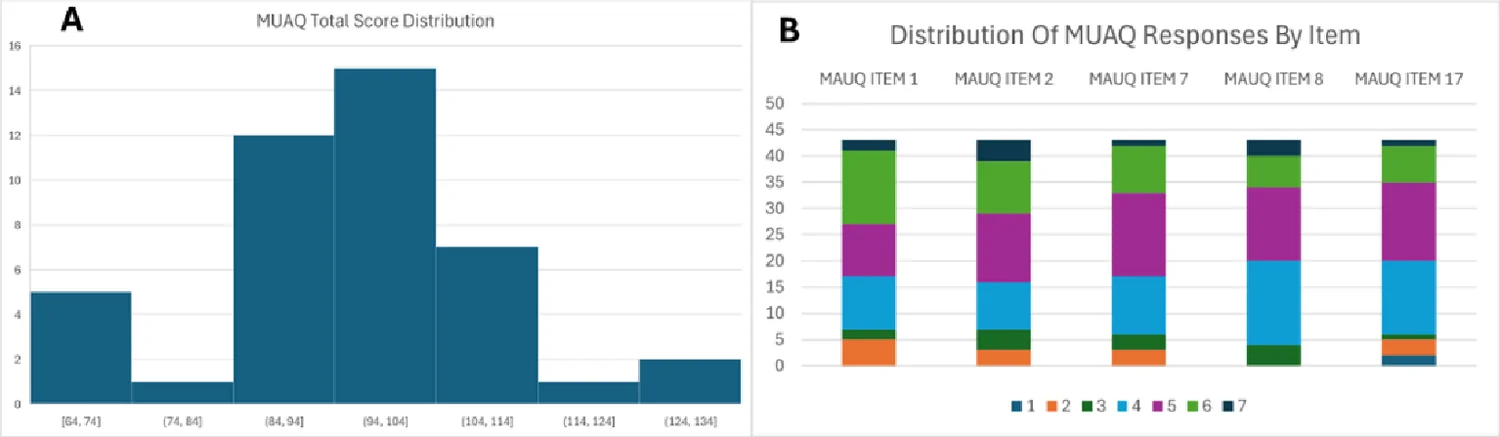
Results of the Mobile Health App Usability Questionnaire (MAUQ) completed by wearable-group patients (*n* = 43). (**A**) Histogram of total MAUQ scores: mean = 95.3 ± 11.4 (86% of responses ≥ 4 on a 7-point Likert scale). (**B**) Stacked bar chart for five representative items-ease of use, learnability, willingness to reuse, overall satisfaction, and perceived effectiveness, showing a predominance of high-range responses (scores 5–7). Higher scores indicate greater usability and satisfaction with the telemonitoring system

The average MAUQ score was 95.3 ± 11.4, indicating generally high user-reported usability, with 86.04% of responses ≥ 4 on the 7-point Likert scale. The analysis focused on five selected items: Item-1 (“The app was easy to use”), Item-2 (“It was easy for me to learn to use the app”), Item-7 (“I would use this app again”), Item-8 (“Overall, I am satisfied with this app”), and Item-17 (“The app helped me manage my health effectively”). These items showed a concentration of responses in the upper range of the scale (scores of 5 to 7), indicating favorable evaluations across multiple usability domains. The MAUQ score distribution and Item analysis are represented in Fig. 4.

## Discussion

The COVID-19 pandemic in 2020 accelerated the adoption of telemedicine and remote patient monitoring, allowing the rapid development of telehealth solutions [11, 18]. In this crisis, wearable devices emerged as practical tools for continuous patient monitoring [19]. Institutions invested heavily in healthcare digitization, including telemedicine, to mitigate the pandemic’s impacts [10, 20]. As a result, telehealth platforms became more widely implemented [21]. This post-pandemic climate provided a favorable backdrop for exploring early-discharge models supported by telemonitoring among surgical patients. Our study evaluated early discharge after major robot-assisted uro-oncologic surgery with wearable-based telemonitoring. Patients in Group W had significantly shorter LoS than Group C (2.8 vs. 4.7 days, *p* < 0.001) due to the protocolized 48-hour earlier discharge. Despite shorter hospitalizations, patients managed via telemonitoring experienced significantly fewer early unscheduled hospital visits than controls (2.32% vs. 25.58%, *p* < 0.01), supporting the feasibility of this postoperative surveillance strategy. Our findings align with the notion that remote postoperative monitoring can detect early complications and potentially prevent unnecessary readmissions [14]. A key outcome of our study was the high user acceptance of the telemonitoring technology, indicating favorable usability and patient satisfaction with the wearable system. Patients found the app and the shirt easy to use and helpful in managing their recovery. Our findings are consistent with those of Breteler et al., who noted that remote vital sign monitoring after early surgical discharge was well received [15]. Careful patient selection for early discharge remains paramount. Frail or medically complex patients have a higher risk of postoperative complications and slower recovery. Our study applied strict inclusion criteria: all patients underwent preoperative cardiopulmonary clearance and had a caregiver at home. An early-discharge protocol following robotic lung lobectomy resulted in zero readmissions or complications, provided each patient met stringent clinical criteria and had caregiver support [20]. These findings were confirmed in a follow-up study [22]. Another pilot study targeting frail cardiac surgery patients demonstrated that vulnerable postoperative patients could be safely managed at home with intensive monitoring; none of the telemonitored frail patients were readmitted within 30 days, versus 14.3% of historical controls [23]. This suggests that some traditionally “high-risk” patients can avoid rehospitalization with the right infrastructure and caregiver and/or family support. In abdominal surgery, Leenen et al. demonstrated that five days of wearable monitoring and daily teleconsultations following colorectal resection effectively detected deviations in vital signs, enabling timely interventions and highlighting how technology can bridge the post-discharge “monitoring gap” [14]. Similarly, Van Ede et al. demonstrated that same-day discharge after bariatric surgery with one week of telemonitoring was non-inferior to standard care, with similar 30-day complication and readmission rates, 61% fewer hospitalization days, and comparable patient satisfaction and pain control [24]. Continuous telemonitoring may provide a “safety net,” enabling earlier discharge with confidence that deterioration will be detected promptly, thereby reducing urgent in-hospital calls [25]. In our protocol, patients wore a multi-sensor shirt linked to a smartphone application, which continuously recorded vital signs and ECG data. Predefined thresholds triggered real-time alerts, immediately transmitted to the clinical monitoring team to evaluate potential deterioration without delay [26]. Over the monitoring period, 68 alerts were generated, including 12 high-priority ones. Most issues were resolved via teleconsultation, with 3 readmissions for events such as AF or hypertensive crises. These readmissions must be distinguished from early voluntary access observed, which were largely precautionary and clinically unnecessary. Notably, the wearable ECG enabled the identification of asymptomatic arrhythmias, underscoring the system’s value in early intervention. Looking to the future, artificial intelligence (AI) has the potential to transform the landscape of wearable-based telemonitoring. Telemonitoring has been used to manage chronic diseases such as heart failure, Chronic Obstructive Pulmonary Disease (COPD), and diabetes [27–30]. In COPD, a Classification and Regression Tree algorithm has identified patients at risk of exacerbation using home-based telehealth data [31]. In our study, thresholds were set to specific values; however, the system was unable to filter out misreads or background noise. In this context, deep learning algorithms can continuously analyze data, learn a patient’s baseline trends, flag subtle deviations that precede clinical symptoms, or filter out spurious readings. Early discharge telemonitoring protocols may have the potential to improve healthcare efficiency by LoS, optimizing bed occupancy, and facilitating patient turnover. The widespread implementation of these protocols could help address hospital capacity constraints and surgical backlogs [32, 33]. Although a systematic review by Raes et al. [34] reported that telemonitoring strategies may be cost-effective, with hospitalization costs reduced by up to $900/patient through shorter LoS and fewer unplanned readmissions, the present study did not include a formal health-economic evaluation. Therefore, the economic implications of this approach in the uro-oncological setting remain to be confirmed in future dedicated cost-effectiveness analyses. Lastly, our study highlights the role of caregivers and the patient experience in this model. Early discharge shifts aspects of care from the hospital to the home, increasing patient and family involvement. The requirement that a caregiver be present reflects the need for a safety net at home. Telemonitoring can empower caregivers by providing them with direct support and guidance. Knowing that the patient is being monitored remotely and can receive professional advice quickly can help alleviate anxiety. However, this model also places additional responsibility on non-healthcare professionals. To mitigate caregiver burden, these models should provide training, clear instructions, and 24/7 assistance for questions or emergencies. In our protocol, caregivers were educated during the preoperative period, and a hotline was available during monitoring. Similarly, other studies have ensured frequent telecheck-ins to support patients at home [22, 35]. Successful implementation of wearable telemonitoring also depends on patient acceptance of digital health technologies. Previous studies have shown that factors such as educational level, familiarity with technology, smartphone use, and awareness of artificial intelligence and wearable applications may significantly influence patients’ willingness to adopt these systems in routine clinical practice [36]. Moving forward, involving caregivers in the design of telemonitoring workflows could further improve the support structure. Current research on wearable telemonitoring in perioperative care is promising yet limited in its scope and generalizability. Many interventions rely on small, single-center studies and often exclude patients without smartphones, limiting applicability beyond the selected cohort [37]. In oncological surgery, trials show feasibility but continue to focus on small populations, and there remains little evidence from continuous wearable sensor data in broad cancer surgery cohorts [38]. The next research phase should involve large, multicenter RCTs across diverse institutions and patient groups to verify if wearable monitoring technology can genuinely enhance outcomes, like faster recovery, earlier complication detection, and fewer readmissions, in real-world settings. AI integration into wearable systems could sharpen detection by learning individual baselines and flagging subtle physiologic deviations that conventional threshold-based monitoring may miss [39]. Furthermore, to date, formal cost analyses are lacking, without rigorous economic evaluation and modeling of system-level impacts, the long-term scaling of telemonitoring programs remains uncertain [40]. By addressing these areas, robust research evidence supporting the widespread adoption of telemonitoring could be developed. This study has several limitations. The relatively small sample size and the requirement for internet access, smartphone availability, and caregiver support may limit the generalizability of this approach, particularly among older patients or individuals with limited digital literacy. Because allocation was based on patient willingness to participate rather than randomization, selection bias cannot be excluded.

Technical issues occurred in 16.27% of patients, potentially affecting real-world implementation despite prompt resolution. Finally, no formal cost-effectiveness analysis was performed, and the long-term economic impact of telemonitoring remains uncertain. Future developments should focus on improving sensor reliability, data integration, and predictive monitoring capabilities.

## Conclusions

In conclusion, our study suggests that early discharge with wearable devices following major robot-assisted uro-oncologic surgery appears to be both feasible and safe in selected patients. The telemonitoring pathway enabled earlier discharge while maintaining continuous postoperative surveillance and was associated with low rates of postoperative complications, readmissions, and unscheduled hospital access. Real-time remote monitoring enabled early detection and management of postoperative anomalies, reinforcing its role as an effective safety net during recovery. While implementing these protocols requires careful patient selection, reliable technology, and system integration, our findings support the use of wearable telemonitoring in recovery pathways. Future research should explore its use in frailer patients, its cost-effectiveness, and its integration with AI analytics to improve postoperative care.

## Supplementary Information

Below is the link to the electronic supplementary material.


Supplementary Material 1


## Data Availability

The data presented in this study are available upon reasonable request to the corresponding author. The data are not publicly available due to privacy/ethical restrictions imposed by the institutions.

## References

[1] Mun SK, Turner JW (1999) Telemedicine: emerging e-medicine. Annu Rev Biomed Eng 1:589–610. 10.1146/annurev.bioeng.1.1.589 11701501

[2] Andersson G (2009) Using the Internet to provide cognitive behaviour therapy. Behav Res Ther 47:175–180. 10.1016/j.brat.2009.01.010 19230862

[3] Fortney JC, Pyne JM, Kimbrell TA, Hudson TJ, Robinson DE, Schneider R, Moore WM, Custer PJ, Grubbs KM, Schnurr PP (2015) Telemedicine-based collaborative care for posttraumatic stress disorder: a randomized clinical trial. JAMA psychiatry 72:58–67. 10.1001/jamapsychiatry.2014.1575 25409287

[4] Kern C, Fu DJ, Kortuem K, Huemer J, Barker D, Davis A, Balaskas K, Keane PA, McKinnon T, Sim DA (2020) Implementation of a cloud-based referral platform in ophthalmology: making telemedicine services a reality in eye care. Br J Ophthalmol 104:312–317. 10.1136/bjophthalmol-2019-314161 31320383 PMC7041498

[5] Wolf JA, Moreau JF, Akilov O, Patton T, English JC 3rd;, Ho J, Ferris LK (2013) Diagnostic inaccuracy of smartphone applications for melanoma detection. JAMA dermatology 149:422–426. 10.1001/jamadermatol.2013.2382 PMC4019431 23325302

[6] Grossman SN, Han SC, Balcer LJ, Kurzweil A, Weinberg H, Galetta SL, Busis NA (2020) Rapid implementation of virtual neurology in response to the COVID-19 pandemic. Neurology 94:1077–1087. 10.1212/wnl.0000000000009677 32358217

[7] Hsieh JC, Li AH, Yang CC (2013) Mobile, cloud, and big data computing: contributions, challenges, and new directions in telecardiology. Int J Environ Res Public Health 10:6131–6153. 10.3390/ijerph10116131 24232290 PMC3863891

[8] Tuveri M, Bassi C, Esposito A, Casetti L, Landoni L, Malleo G, Marchegiani G, Paiella S, Fontana M, De Pastena M et al (2022) Bioethics in an oncological surgery unit during the COVID-19 pandemic: the Verona experience. Updates Surg 74:1247–1252. 10.1007/s13304-022-01279-5 35298787 PMC8927519

[9] Chen G, Shen S, Tat T, Zhao X, Zhou Y, Fang Y, Chen J (2022) Wearable respiratory sensors for COVID-19 monitoring. View (Beijing China) 3:20220024. 10.1002/viw.20220024 36710943 PMC9874505

[10] Parretti C, La Regina M, Tortu C, Candido G, Tartaglia R (2023) Telemedicine in Italy, the starting point. Intern Emerg Med 18:949–951. 10.1007/s11739-022-03176-6 36539605 PMC9767849

[11] Parretti C, Tartaglia R, La Regina M, Venneri F, Sbrana G, Mandò M, Barach P (2022) Improved FMEA Methods for Proactive Health Care Risk Assessment of the Effectiveness and Efficiency of COVID-19 Remote Patient Telemonitoring. Am J Med quality: official J Am Coll Med Qual 37:535–544. 10.1097/jmq.0000000000000089 PMC9622365 36250651

[12] Tuckson RV, Edmunds M, Hodgkins ML, Telehealth (2017) N Engl J Med 377:1585–1592. 10.1056/NEJMsr1503323 29045204

[13] Huhn S, Axt M, Gunga HC, Maggioni MA, Munga S, Obor D, Sié A, Boudo V, Bunker A, Sauerborn R et al (2022) The Impact of Wearable Technologies in Health Research: Scoping Review. JMIR mHealth uHealth 10:e34384. 10.2196/34384 35076409 PMC8826148

[14] Leenen JPL, Ardesch V, Patijn G (2023) Remote Home Monitoring of Continuous Vital Sign Measurements by Wearables in Patients Discharged After Colorectal Surgery: Observational Feasibility Study. JMIR Perioper Med 6:e45113. 10.2196/45113 37145849 PMC10199380

[15] Breteler MJM, Numan L, Ruurda JP, van Hillegersberg R, van der Horst S, Dohmen DAJ, van Rossum MC, Kalkman CJ (2020) Wireless Remote Home Monitoring of Vital Signs in Patients Discharged Early After Esophagectomy: Observational Feasibility Study. JMIR Perioper Med 3:e21705. 10.2196/21705 33393923 PMC7728408

[16] Mangiameli G, Bottoni E, Tagliabue A, Giudici VM, Crepaldi A, Testori A, Voulaz E, Cariboni U, Re Cecconi E, Luppichini M et al (2024) Early Hospital Discharge on Day Two Post-Robotic Lobectomy with Telehealth Home Monitoring. J Clin Med 13. 10.3390/jcm13206268 PMC11508382 39458218

[17] Zhou L, Bao J, Setiawan IMA, Saptono A, Parmanto B (2019) The mHealth App Usability Questionnaire (MAUQ): Development and Validation Study 7:e11500. 10.2196/11500 PMC6482399 30973342

[18] Gareev I, Gallyametdinov A, Beylerli O, Valitov E, Alyshov A, Pavlov V, Izmailov A, Zhao S (2021) The opportunities and challenges of telemedicine during COVID-19 pandemic. Front Biosci 13:291–298. 10.52586/e885 34937315

[19] Channa A, Popescu N, Skibinska J, Burget R (2021) The Rise of Wearable Devices during the COVID-19 Pandemic: A Systematic Review. Sensors 21. 10.3390/s21175787 PMC8434481 34502679

[20] Colbert GB, Venegas-Vera AV, Lerma EV (2020) Utility of telemedicine in the COVID-19 era. Rev Cardiovasc Med 21:583–587. 10.31083/j.rcm.2020.04.188 33388003

[21] Haider Z, Aweid B, Subramanian P, Iranpour F (2022) Telemedicine in orthopaedics during COVID-19 and beyond: A systematic review. J Telemed Telecare 28:391–403. 10.1177/1357633x20938241 32762270 PMC9124641

[22] Mangiameli G, Bottoni E, Tagliabue A, Giudici VM, Crepaldi A, Testori A, Voulaz E, Cariboni U, Re Cecconi E, Luppichini M et al (2024) Early Hospital Discharge on Day Two Post-Robotic Lobectomy with Telehealth Home Monitoring. 13:6268 10.3390/jcm13206268 PMC11508382 39458218

[23] Sarkar S, MacLeod J, Hassan A, Brunt KR, Palmer K, Légaré JF (2022) Enhanced Telehealth Home-Monitoring Intervention for Vulnerable and Frail Patients after Cardiac Surgery (THE-FACS Pilot Intervention Study). BMC Geriatr 22:836. 10.1186/s12877-022-03531-4 36333652 PMC9636804

[24] van Ede ES, Scheerhoorn J, Buise MP, Bouwman RA, Nienhuijs SW (2023) Telemonitoring for perioperative care of outpatient bariatric surgery: Preference-based randomized clinical trial. PLoS ONE 18:e0281992. 10.1371/journal.pone.0281992 36812167 PMC9946229

[25] Leenen JPL, Ardesch V, Kalkman CJ, Schoonhoven L, Patijn GA (2024) Impact of wearable wireless continuous vital sign monitoring in abdominal surgical patients: before-after study. BJS open 8. 10.1093/bjsopen/zrad128 PMC10794900 38235573

[26] Clark M, Bailey S (2023) CADTH Horizon Scans. Single-Use Wearable Wireless Sensors for Vital Sign Monitoring: CADTH Horizon Scan. Canadian Agency for Drugs and Technologies in Health: Ottawa (ON) 38320082

[27] Bolton CE, Waters CS, Peirce S, Elwyn G (2011) Insufficient evidence of benefit: a systematic review of home telemonitoring for COPD. J Eval Clin Pract 17:1216–1222. 10.1111/j.1365-2753.2010.01536.x 20846317

[28] Mohktar MS, Redmond SJ, Antoniades NC, Rochford PD, Pretto JJ, Basilakis J, Lovell NH, McDonald CF (2015) Predicting the risk of exacerbation in patients with chronic obstructive pulmonary disease using home telehealth measurement data. Artif Intell Med 63:51–59. 10.1016/j.artmed.2014.12.003 25704112

[29] Inglis SC, Clark RA, McAlister FA, Stewart S, Cleland JG (2011) Which components of heart failure programmes are effective? A systematic review and meta-analysis of the outcomes of structured telephone support or telemonitoring as the primary component of chronic heart failure management in 8323 patients: Abridged Cochrane Review. Eur J Heart Fail 13:1028–1040. 10.1093/eurjhf/hfr039 21733889

[30] Polisena J, Tran K, Cimon K, Hutton B, McGill S, Palmer K (2009) Home telehealth for diabetes management: a systematic review and meta-analysis. Diabetes Obes Metab 11:913–930. 10.1111/j.1463-1326.2009.01057.x 19531058

[31] Kuziemsky C, Maeder AJ, John O, Gogia SB, Basu A, Meher S, Ito M (2019) Role of Artificial Intelligence within the Telehealth Domain. Yearb Med Inform 28:35–40. 10.1055/s-0039-1677897 31022750 PMC6697552

[32] Oliveira M, Bélanger V, Ruiz A, Santos D (2023) A systematic literature review on the utilization of extended operating room hours to reduce surgical backlogs. Front public health 11:1118072. 10.3389/fpubh.2023.1118072 37124824 PMC10133497

[33] Guerrieri R, Rovati L, Dell’Oglio P, Galfano A, Ragazzoni L, Aseni P (2021) Impact of the COVID-19 Pandemic on Urologic Oncology Surgery: Implications for Moving Forward. J Clin Med 11. 10.3390/jcm11010171 PMC8745246 35011911

[34] Raes S, Prezzi A, Willems R, Heidbuchel H, Annemans L (2024) Investigating the Cost-Effectiveness of Telemonitoring Patients With Cardiac Implantable Electronic Devices: Systematic Review. J Med Internet Res 26:e47616. 10.2196/47616 38640471 PMC11069092

[35] Bottoni E, Mangiameli G, Testori A, Piccioni F, Giudici VM, Voulaz E, Ruggieri N, Dalla Corte F, Crepaldi A, Goretti G et al (2023) Early Hospital Discharge on Day Two Post Robotic Lobectomy with Telehealth Home Monitoring: A Pilot Study. 15:1146 10.3390/cancers15041146 PMC9954553 36831489

[36] Manolitsis I, Tzelves L, Feretzakis G, Kalles D, Verykios VS, Katsimperis S, Anastasiou A, Koutsouris D, Kosmidis T, Deliveliotis C et al (2023) Acceptance of Artificial Intelligence in Supporting Cancer Patients. Stud Health Technol Inf 305:572–575. 10.3233/shti230561 37387095

[37] Knight SR, Ng N, Tsanas A, McLean K, Pagliari C, Harrison EM (2021) Mobile devices and wearable technology for measuring patient outcomes after surgery: a systematic review. npj Digit Med 4:157. 10.1038/s41746-021-00525-1 34773071 PMC8590052

[38] Sun V, Xiao Y, Li S, Palmer J, Tribby CP, Crane TE, Melstrom L, Dellinger T, Yuh B, Raz D et al (2025) Comparative effectiveness of remote perioperative telemonitoring in cancer surgery: a randomized trial. npj Digit Med 8:555. 10.1038/s41746-025-01961-z 40877442 PMC12394556

[39] Khan MM, Shah N (2025) AI-driven wearable sensors for postoperative monitoring in surgical patients: A systematic review. Comput Biol Med 196:110783. 10.1016/j.compbiomed.2025.110783 40680357

[40] Amin T, Mobbs RJ, Mostafa N, Sy LW, Choy WJ (2021) Wearable devices for patient monitoring in the early postoperative period: a literature review. Mhealth 7:50. 10.21037/mhealth-20-131 34345627 PMC8326951

