# Supplementary material for: Feasibility and safety of early discharge after robot-assisted uro-oncologic surgery using wearable telemonitoring
Source: J Robot Surg. 2026 Aug 31;20(1):913. doi: 10.1007/s11701-026-03696-9 (PMC13529836; doi:10.1007/s11701-026-03696-9)
Supplement: Supplementary file 1 [file 11701_2026_3696_MOESM1_ESM.pdf]

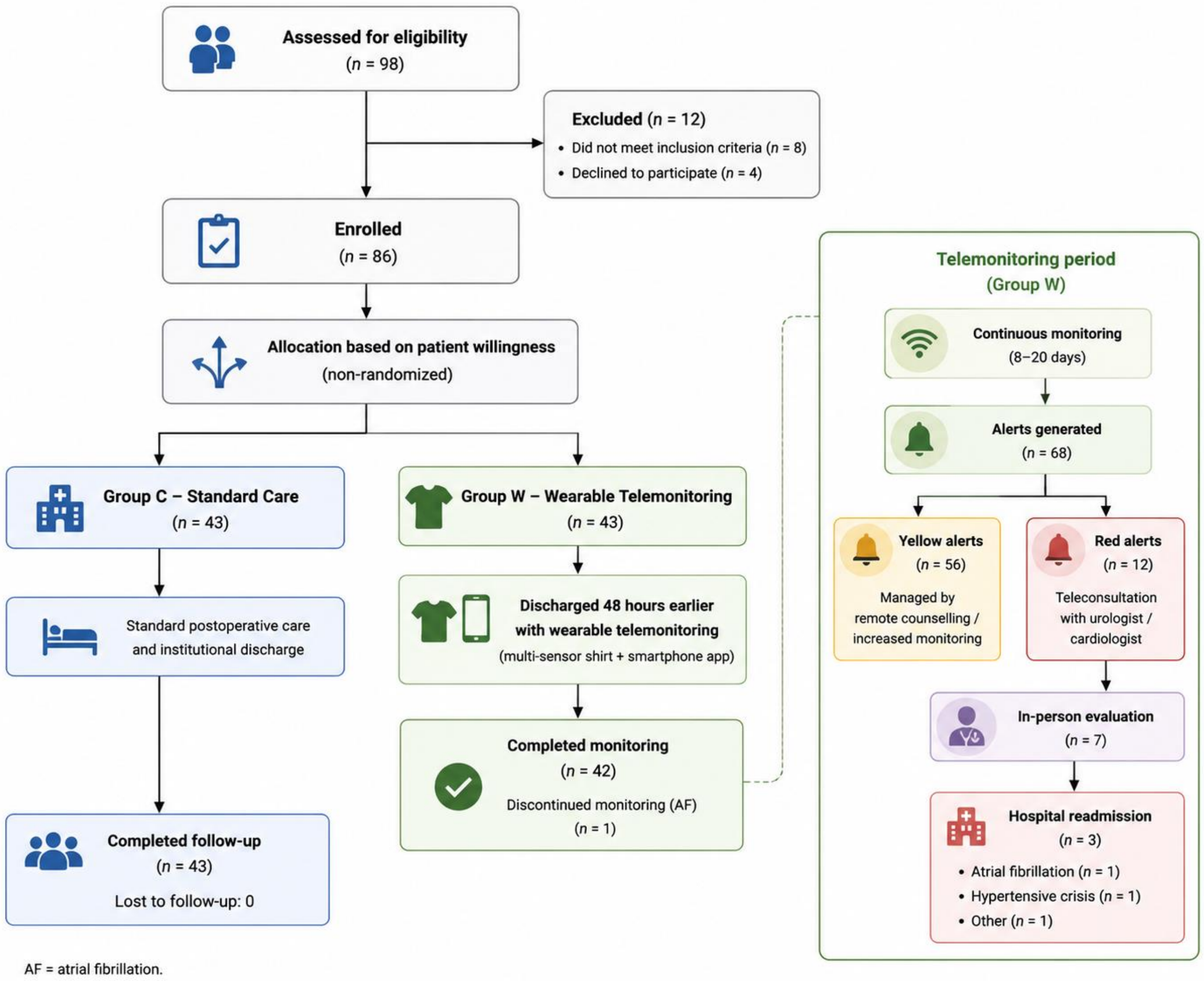

Supplementary Figure S1. Flow diagram of patient enrollment, group allocation, completion of telemonitoring, alerts, hospital evaluations, and readmissions.
